# Long-Term Anticoagulation in Secondary Ischemic Stroke Prevention: The Prospective Multicenter RESTAIC Registry

**DOI:** 10.3389/fneur.2020.575634

**Published:** 2020-10-19

**Authors:** Raquel Gutiérrez-Zúñiga, Ricardo Rigual, Gabriel Torres-Iglesias, Sara Sánchez-Velasco, María Alonso de Leciñana, Jaime Masjuan, Rodrigo Álvarez Velasco, Inmaculada Navas, Laura Izquierdo-Esteban, José Fernández-Ferro, Jorge Rodríguez-Pardo, Gerardo Ruiz-Ares, Gustavo Zapata-Wainberg, Blanca Fuentes, Exuperio Díez-Tejedor

**Affiliations:** ^1^Neurology Department and Stroke Centre, Hospital La Paz Institute for Heath Research-IdiPAZ, La Paz Univerisity Hospital, Universidad Autónoma de Madrid, Madrid, Spain; ^2^Departamento de Medicina, Facultad de Medicina, Servicio de Neurología, H Universitario Ramón y Cajal, Universidad de Alcalá, IRYCIS, Madrid, Spain; ^3^Neurology Department, Hospital Universitario Fundación Jiménez Díaz, Madrid, Spain; ^4^Neurology Department, Hospital Universitario Príncipe de Asturias, Madrid, Spain; ^5^Neurology Department and Stroke Unit, Hospital Universitario Rey Juan Carlos, Madrid, Spain; ^6^Neurology Department, Hospital Universitario La Princesa, Madrid, Spain

**Keywords:** secondary stroke prevention, anticoagulant drugs, stroke recurrence, hemorrhage risk, multicenter registry

## Abstract

**Background and Objective:** Oral anticoagulation (OAC) for secondary stroke prevention is recommended in atrial fibrillation (AF) and other sources of cardioembolic stroke. Our objectives were to explore the differences in ischemic and hemorrhagic events when using OAC for secondary stroke prevention according to the type of anticoagulant treatment and to analyze the number and reasons for OAC switches during long-term follow-up.

**Methods:** Ischemic stroke (IS) patients who were discharged on OAC for secondary stroke prevention from January 2014 to October 2017 were recruited in a prospective, multicenter, hospital-based registry. Follow-up at 3 months was scheduled at the outpatient clinic with subsequent annual phone interviews for 3 years. Patients were classified into three study groups according to OAC at discharge: Vitamin K antagonist (VKA), Factor Xa inhibitor (FXa), or direct thrombin inhibitor (DTI). We compared stroke recurrences, intracranial hemorrhage, major bleeding, and all-cause mortality during the follow-up. We recorded any switches in OAC and the main reasons for the change.

**Results:** A total of 241 patients were included. An anticoagulant was indicated in the presence of a source of cardioembolic stroke in 240 patients (99.6%) and lupus plus antiphospholipid syndrome in one patient. Up to 86 patients (35.6%) were on OAC before the index stroke; in 71 (82.5%) of them, this was VKA. At hospital discharge, 105 were treated with FXa (43.8%), 96 with VKA (39.6%), and 40 with DTI (16.6%). The cumulative incidences at 3 years were 17% for stroke recurrence, 1.6% for intracranial hemorrhage, 4.9% for major hemorrhage, and 22.8% for all-cause mortality, with no differences among the OAC groups in any outcomes. During the follow-up, 40 OAC switches were recorded (63% of them to FXa), mostly due to stroke recurrence.

**Conclusion:** Long-term OAC in secondary stroke prevention is associated with a lower frequency of bleeding complications than stroke recurrences. No differences between anticoagulant drugs were found in any of the analyzed outcomes. The main cause for OAC switch during follow-up was stroke recurrence.

## Introduction

Current guidelines recommend oral anticoagulation (OAC) for secondary stroke prevention in atrial fibrillation (AF) and other sources of cardioembolic stroke (1, 2). Vitamin K antagonists (VKA) were the only treatments available until the publication of several clinical trials showing the non-inferiority of direct oral anticoagulants (DOACs) in the prevention of stroke and peripheral embolisms in patients with non-valvular AF, with the advantage of lower risk of intracranial bleeding complications (3). Therefore, since then, the prescription of DOACs has increased for primary stroke prevention, with a parallel decrease in VKA use (4, 5). Several real-life registries found that DOACs are safe and effective drugs for primary stroke prevention in AF patients (6–9). Moreover, evidence supporting the efficacy and safety of DOACs comes from randomized clinical trials that recruited AF patients at high risk of stroke but with only about 19–52% of patients with previous stroke or transient ischemic attack (TIA) (10–13). All these trials have published the results of this subgroup of patients showing consistently beneficial results in secondary stroke prevention (14–17). Taking into account that stroke patients are at high risk both of ischemic and hemorrhagic strokes, and prevention of both are the main objectives in this specific population, it seems necessary to monitor the effects of OAC in non-selected patients in clinical practice. Several registries have been published in recent years to study the use of the various oral anticoagulant treatments in stroke patients (18–26), mainly evaluating clinical outcomes such as stroke recurrence, cerebral hemorrhage, or functional outcome. Other published registries were focused on the use of radiological markers such as cerebral microbleeds or white matter hyperintensities in order to stratify the hemorrhagic risk in this specific population (27, 28). Also, individual patient data analysis of seven prospective cohort studies has been recently published addressing the outcomes in patients with AF who received anticoagulation treatment in secondary prevention after stroke (29).

However, the “real-world” long-term outcomes of stroke patients receiving OAC for any indication (not restricted to AF) in secondary stroke prevention and the number and reasons for OAC switches during follow-up are not well-known, missing relevant information for the clinician.

Our aims were to evaluate the effects of OAC in long-term outcomes in a secondary prevention clinical setting and to analyze the number of, and reasons for, switches among various anticoagulants.

## Methods

### Study Design

The RESTAIC (acronym corresponding to the Spanish name *REgistro cl*í*nico para el Seguimiento de Tratamiento Anticoagulante en prevención secundaria tras un Infarto Cerebral; Clinical registry for the follow-up of anticoagulant treatment in secondary prevention following cerebral infarction*) was a multicenter, prospective, observational study supported by the IdiPAZ research foundation, conducted in six departments of neurology in Madrid, Spain (see the participant list in the Acknowledgments section). Ischemic stroke (IS) patients who were discharged on OAC were recruited from January 2014 to October 2017. The prescription of OAC was the decision of the treating physician. The study protocol did not provide a recommendation for the prescription of one anticoagulant drug over other.

The inclusion criteria were 18 years old or older (no upper age limit), admitted with IS (TIA or cerebral infarction) with indication of anticoagulation for secondary stroke prevention (source of cardioembolic stroke or another indication), and able to understand and sign the informed consent (in the event of a patient's incapacity, informed consent could be signed by a relative). The exclusion criteria were participation in another observational study or in clinical trials, severe disease with life expectancy of <3 years, and patients who were not discharged on OAC. The follow-up period after inclusion was up to 3 years. The first assessment was during the hospital admission for the index stroke, and the follow-up visits were scheduled as follows: one visit at 3 months at the outpatient clinic and subsequent annual telephone interviews for 3 years. These consultations were carried out centrally from the main study center.

The study was approved by the ethics committee of each participating hospital. The study was approved by the Spanish Agency of Medicines and Medical Devices (*Agencia Española de Medicamentos y Productos Sanitarios*).

#### Outcomes

The primary outcomes were ischemic stroke recurrence, intracranial hemorrhage, major bleeding [defined according to the International Society on Thrombosis and Haemostasis (ISTH)] (30), and all-cause mortality. The secondary outcomes were clinically relevant non-major bleeding (CRNMB), defined according to the ISTH criteria (31): minor bleeding, peripheral embolism, and vascular-related mortality (ischemic stroke, intracranial hemorrhage, acute myocardial infarction, acute heart failure, arrhythmia, and pulmonary thromboembolism). We also recorded any switches in OAC during follow-up and the main reasons for the change (stroke recurrence, intracranial hemorrhage, major bleeding, CRNMB or minor bleeding, peripheral embolism, labile INR in those treated with VKA, and other reasons not related to direct complications of anticoagulation).

### Data Collection

A specific electronic case report form was developed for this study on enrollment: demographic variables (age, sex, educational level, and current occupational activity); previous history of hypertension, diabetes mellitus (DM), tobacco smoking, previous IS (other than index stroke), or TIA, the presence of a source of cardioembolic stroke (AF, prosthetic valve, other sources of cardioembolic stroke: other non-prosthetic valvulopathy, intracardiac thrombus, ventricular akinesia, left ventricular aneurysm, left ventricular ejection fraction ≤35%, patent foramen ovale, atrial septal aneurysm, dilated cardiomyopathy, chronic heart failure, endocarditis, and atrial myxoma). OAC prior to index stroke, Charlson comorbidity index (CCI) (32), modified Rankin Scale (mRS), CHA2-DS2-VASc and HAS-BLED scores at discharge were recorded.

Additional variables relating to the index stroke were recorded: main indication of OAC (source of cardioembolic stroke or another noncardiac indication for anticoagulation) and type of anticoagulant at discharge: Vitamin K antagonists (VKA), Factor Xa inhibitors (FXa), or direct thrombin inhibitors (DTI). At the follow-up visits, occurrences of any primary or secondary outcomes were recorded.

### Statistical Analysis

The statistical analysis was performed using SPSS 26.0 for Windows (SPSS Inc., Chicago, IL). First, we carried out a descriptive analysis of the total cohort. Categorical variables were presented as proportions and continuous variables as the mean and standard deviation (SD) or median and interquartile range (IQR).

Baseline and demographic characteristics were then analyzed and compared among the various types of anticoagulants at discharge: VKA, FXa, and DTI. Categorical variables were compared using *X*^2^ or Fisher's exact test where appropriate, and quantitative variables were compared using ANOVA test and underwent *post hoc* adjustment using the Bonferroni test. We performed an intention-to-treat analysis.

We calculated the cumulative incidence for the primary and secondary outcomes at 3-years follow-up, and those were compared using a Kaplan–Meier and log rank analysis, according to the anticoagulant type at discharge (VKA vs. FXa vs. DTI). Moreover, we performed a multivariate Cox regression analysis by the “enter” method, adjusting by sex, age, Charlson comorbidity index, and previous stroke.

Finally, a descriptive analysis of switches among anticoagulant drugs and main reasons for them during the 3-years follow-up was carried out. All tests were two-tailed; significance was assumed when *p* < 0.05

## Results

**Figure 1:**
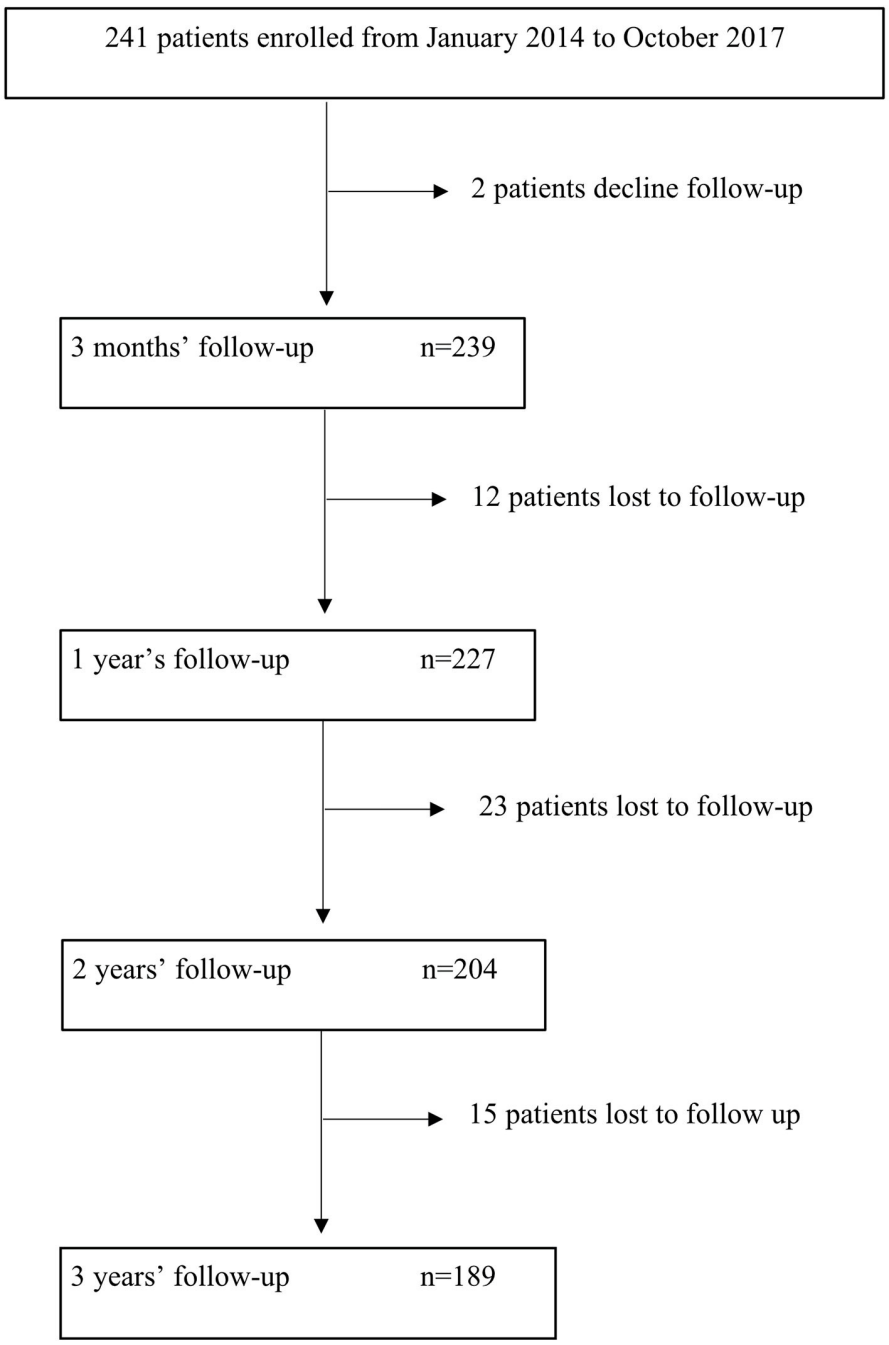
Flow chart of study enrollment.

A total of 241 patients were enrolled, but 52 patients dropped out of the study during follow-up: two patients refused to continue with the telephone interviews, and 50 patients could not be contacted after several attempts (Figure 1). The study database was closed in April 2020, after completion of a 3 years follow-up in 189 patients. The mean time of follow-up was 31.2 months. The total patient-years of follow up was 1,261.75 patients/year. The patients who did not complete the entire follow-up were younger (mean age 71.6 vs. 74.78 years) and had a lower rate of dyslipidemia (48 vs. 65%), with no other differences in demographics and baseline characteristics.

The demographics and baseline characteristics of the cohort are described in Table 1. Two hundred forty patients had a source of cardioembolic stroke as the main indication for OAC, and only one patient had a non-cardioembolic disease (lupus plus antiphospholipid syndrome). At hospital discharge, 105 patients (43.6%) received FXa, 96 (39.8%) VKA, and 40 (16.6%) DTI treatment. In general, patients given FXa were older, with a higher proportion of hypertension and higher scores on the CHA2-DS2-VASc and HAS-BLED scales. VKA-treated patients had a higher proportion of non-AF sources of cardioembolic stroke as a main reason for anticoagulation than the other groups.

**Table 1:** Demographic characteristics of the total cohort.

**Demographic data, *n* (%)**	**Total cohort** **N = 241**	**VKA** **N = 96** **(39.8)**	**FXa** ***N* = 105** **(43.6)**	**DTI** **N = 40** **(16.6)**	p*
Sex male, *n* (%)	132 (54.8)	59 (61.5)	53 (50.5)	20 (50)	0.23
Age; years, mean (±SD)	74.1 (11.8)	70.2 (12.5)	77.8^¶^ (10.2)	73.4(10.9)	<0.001
**Educational level**, ***n*** **(%)**					
Unable to read or write	15 (6.2)	10 (10.4)	4 (3.8)	1 (2.5)	0.20
Read and write only	40 (16.5)	11 (11.5)	21 (19.8)	8 (20)	
Primary/secondary education	110 (45.4)	47 (49)	49 (46.7)	18 (45)	
Higher non-university studies	24 (9.9)	14 (14.6)	8 (7.6)	2 (5)	
University/college degree	49 (20.2)	14 (14.6)	23 (22.6)	11 (27.5)	
**Occupational activity**, ***n*** **(%)**					
Not working	40 (16.5)	14 (14.6)	21 (20)	5 (12.5)	0.08
Working	34 (14)	21 (21.9)	6 (5.7)	7 (17.5)	
Incapacity	9 (3.7)	6 (6.3)	2 (2)	1 (2.5)	
Retired	159 (65.7)	55 (57.3)	76 (72.4)	27 (67.5)	
Hypertension, *n* (%)	189 (78.4)	67 (69.8)	91 (86.7) ^¶^	31 (77.5)	0.01
Diabetes mellitus, *n* (%)	61 (25.3)	18 (18.8)	31 (29.5)	12 (30)	0.16
Dyslipidemia, *n* (%)	148 (61.4)	60 (62.5)	65 (61.9)	23 (57.5)	0.85
Chronic kidney disease, *n* (%)	17 (7.1)	7 (7.3)	8 (7.6)	2 (5)	0.87
Tobacco smoking, *n* (%) *n* (%)	35 (14.5)	18 (18.8)	12 (11.4)	5 (12.5)	0.31
Previous TIA or IS, *n* (%)	63 (26.1)	17 (17.7)^¶^	32 (30.5)	14 (35)	0.04
Previous peripheral embolism, *n* (%)	5 (2.1)	2 (2.1)	2 (1.9)	1 (2.5)	0.97
**Previous source of cardioembolic stroke**, ***n*** **(%)**					
AF	171 (70.7)	44 (45.8)^¶^	92 (86.8)	35 (87.5)	<0.001
Prosthetic valve	14 (5.8)	14 (14.6)	0	0	
Others	27 (11.2)	26 (27.1)	1 (0.9)	0	-
**Previous anticoagulant**, ***n*** **(%)**					
VKA	71 (29.5)	24 (25)	36 (34.3)	11 (27.5)	0.22
FXa	12 (5)	0	8 (7.6)	4 (10)	-
DTI	3 (1.2)	0	2 (1.9)	1 (2.5)	-
**Main indication for anticoagulation at discharge**, ***n*** **(%)**					
AF	188 (78)	44 (45.8)^¶^	104 (99)	40 (100)	<0.001
Prosthetic valve	14 (5.8)	14 (14.6)	0	0	-
Other source of cardioembolic stroke	38 (15.8)	38 (39.6)	1 (1)	0	-
Other non-cardiac conditions	1 (0.4)	0		0	-
CCI at discharge; median (IQR)	1 (0–2)	2 (0–3)	1 (0–2)	1(0–2)	0.36
mRS at discharge; median (IQR)	1 (0–2)	1 (0–2)	1 (0–3)	1 (0–3)	0.03
CHA2-DS2-VASc at discharge; median (IQR)	5 (4–6)	5^¶^ (3–6)	6^¶^ (5–7)	5 (4–7)	<0.001
HAS-BLED at discharge; median (IQR)	2 (2–3)	2^¶^ (2–3)	3^¶^ (2–3)	2 (2–3)	<0.01

* Comparative analysis of the 3 anticoagulant groups: VKA, FXa and DTI.

¶ *Statistically significant differences after Bonferroni correction. CCI, Charlson Comorbidity Index; IS, Ischaemic stroke; TIA, Transient ischaemic attack; AF, Atrial fibrillation; VKA, Vitamin K antagonist; FXa, Factor Xa inhibitor; DTI, Direct thrombin inhibitor*.

**Figure 2:**
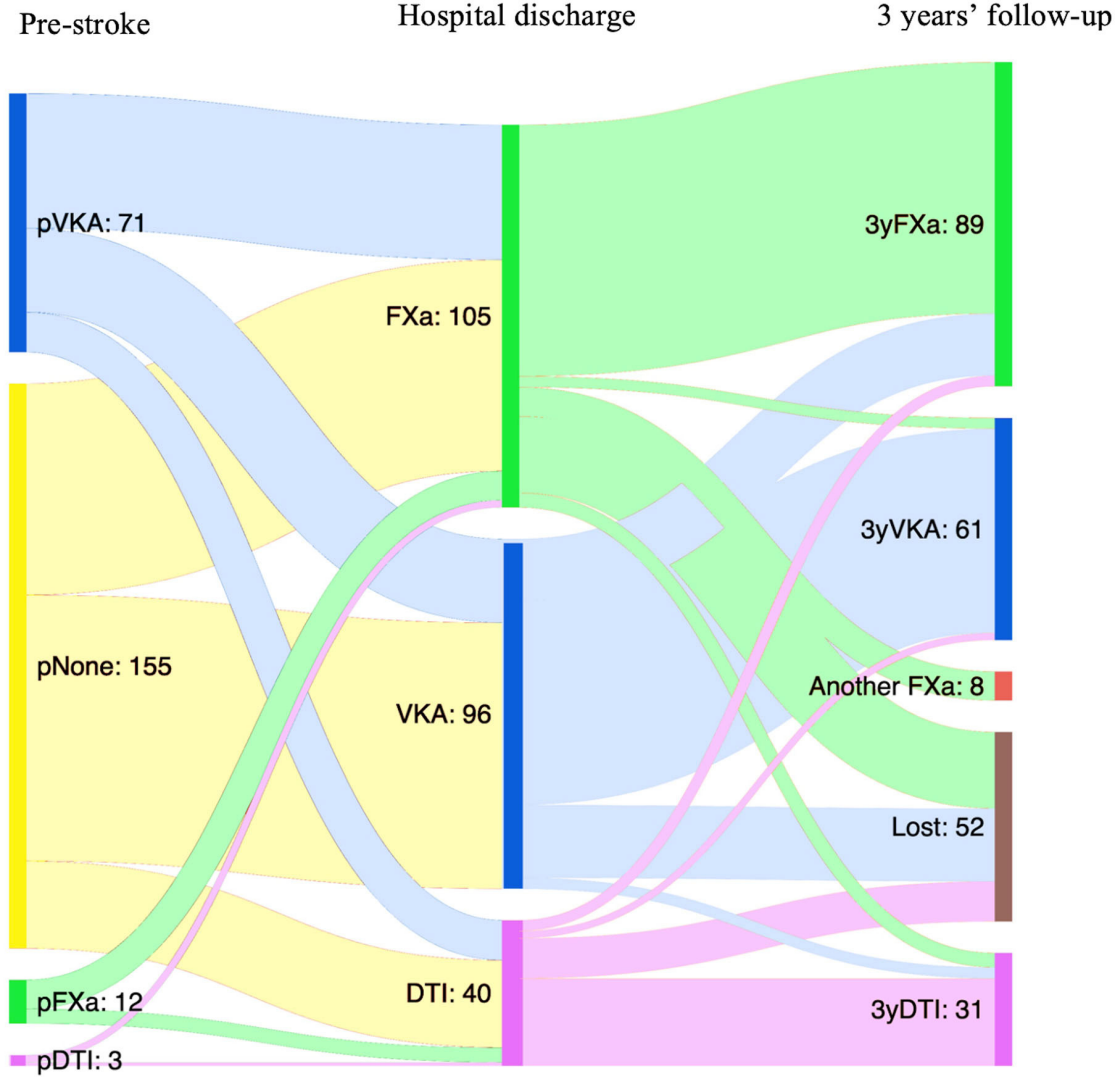
Type of anticoagulant treatment at the time of index stroke, at hospital discharge, and at 3 years of follow-up. VKA, vitamin K antagonist; FXa, Factor Xa inhibitor; DTI, direct thrombin inhibitor; pNone, no previous anticoagulant treatment; pVKA, previously treated with vitamin K antagonist; pFXa, previously treated with Factor Xa inhibitor; pDTI, previously treated with direct thrombin inhibitor; 3yVKA, VKA at 3 years of follow-up; 3yFXa, Factor Xa inhibitor at 3 years of follow-up; 3yDTI, direct thrombin inhibitor at 3 years of follow-up.

For each participant, OAC before the index stroke and after discharge is shown in Figure 2. Eighty-six patients (35.6% of the total cohort) were on anticoagulation before the index stroke. Of these, 71 (82.5%) were on VKA treatment. Noticeably, 61.1% of the INR values were below the recommended range at admission (according to specific indication: non-valvular AF or mechanical prosthetic valve).

Another notable finding is that 63 patients (26% of the total cohort) had already had a stroke before the index stroke. Only 32 (50% of them) were on anticoagulation treatment before the index stroke, the majority of them receiving VKA (*n* = 23; 71.87%). Eighteen patients changed to DOAC, and only five patients, previously VKA anticoagulated in secondary stroke prevention, maintained VKA at discharge after the index stroke—four of them because of the presence of a contraindication to a DOAC (two patients with prosthetic valves, one patient with valvular AF, and one patient with ventricular akinesia). None of the patients previously treated with DOAC changed to VKA for the index stroke.

**Figure 3:**
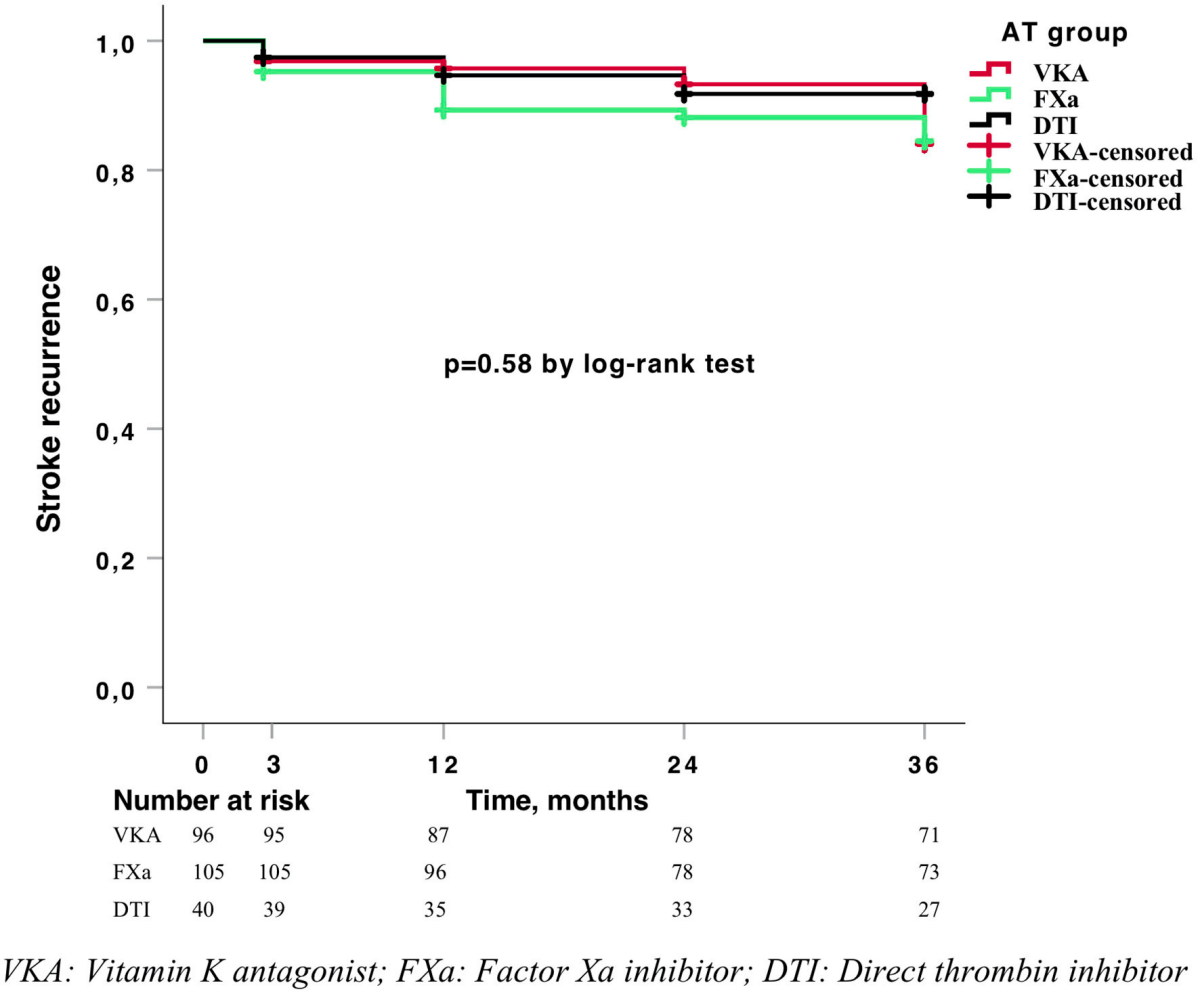
Kaplan–Meier curve for stroke recurrence.

**Figure 4:**
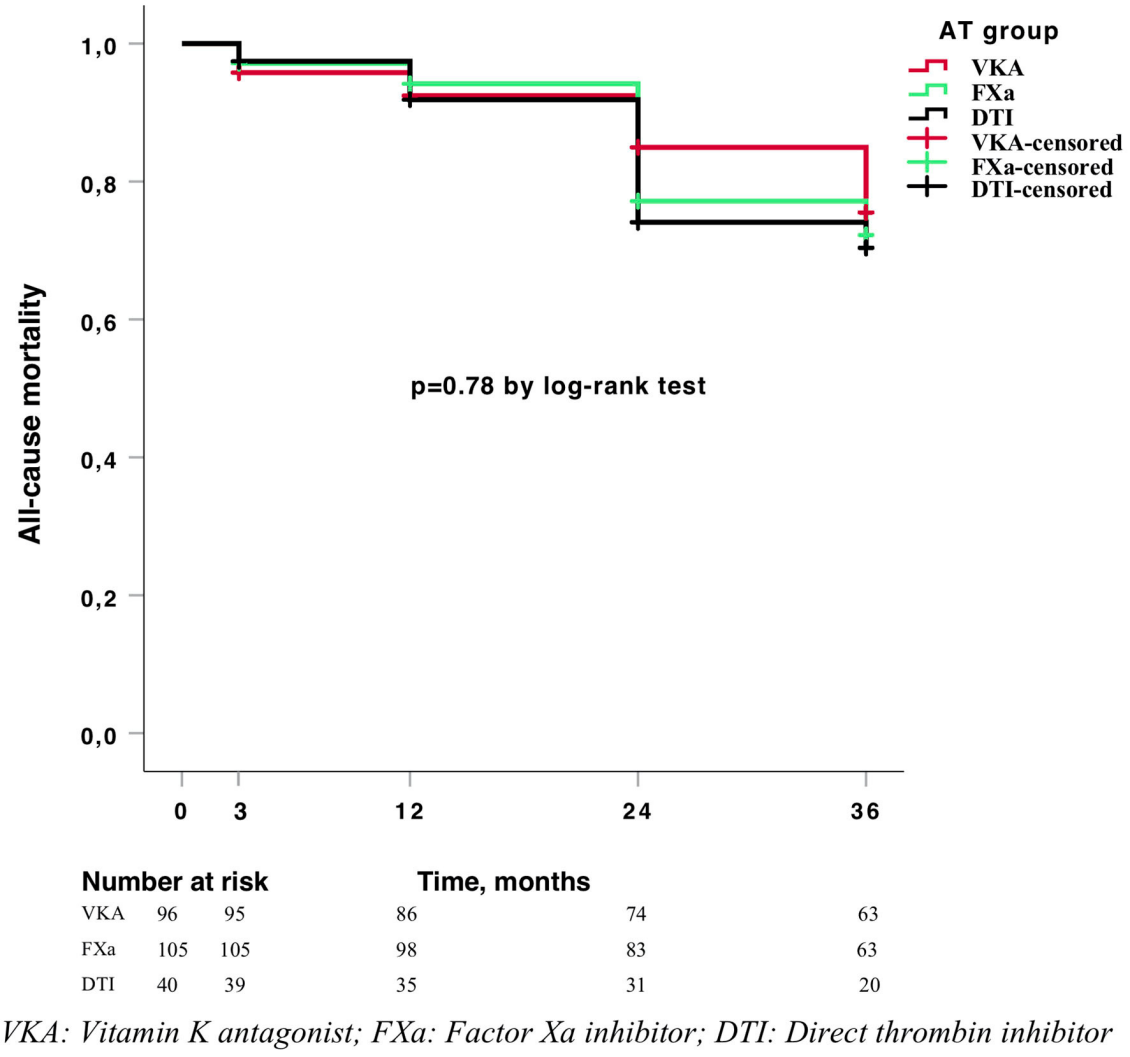
Kaplan–Meier curve for mortality.

The results for the primary and secondary outcomes are shown in Table 2. Overall, we did not find any differences in outcomes between the treatment groups. The cumulative incidence at 3 years was 17% for stroke recurrence, 1.6% for intracranial hemorrhage, 4.9% for major bleeding, and 22.8% for all-cause mortality, with no differences between anticoagulant groups in the Kaplan–Meier analysis (Figures 3, 4). Regarding the baseline characteristics, patients with a previous stroke presented higher stroke recurrence (22.2 vs. 9.5%, *p* = 0.01) and overall mortality (33.3 vs. 19.1%, *p* = 0.021) after a 3-years follow-up than patients without a previous history of stroke. The long-term risk of stroke recurrence, intracranial hemorrhage, major bleeding, and all-cause mortality was similar after adjustment for age, sex, comorbidity, and previous stroke (Table 2).

**Table 2:** Number of events, cumulative incidence, log-rank analysis, and hazard ratio (HR) for primary and secondary outcomes.

	**Number of events**	**Cumulative incidence**	**Log rank**	***P***	**Adjusted HR (95% CI)**
**STROKE RECURRENCE**					
VKA	18				Ref.
FXa	20				0.94 (0.41–2.21)
DTI	4				0.68 (0.18–2.46)
Total	42	17%	1.07	0.58	
**INTRACRANIAL HEMORRHAGE**					
VKA	2				Ref.
FXa	1				0.43 (0.03–5.68)
DTI	1				1.45 (0.12–17.08)
Total	4	1.6%	61	0.73	
**MAJOR BLEEDING**					
VKA	6				Ref.
FXa	4				1.02 (0.06–15.53)
DTI	2				2.54 (0.17–37.17)
Total	12	4.9%	0.62	0.73	
**ALL-CAUSE MORTALITY**					
VKA	20				Ref.
FXa	25				0.44 (0.12–1.66)
DTI	10				0.83 (0.16–4.22)
Total	55	22%	0.43	0.78	
**CRNMH**					
VKA	15				Ref.
FXa	18				0.57 (0.15–2.02)
DTI	4				0.49 (0.08–2.78)
Total	35	14.4%	0.54	0.77	
**MINOR BLEEDING**					
VKA	17				Ref.
FXa	7				0.53 (0.13–2.16)
DTI	4				0.34 (0.04–2.97)
Total	28	11.6%	5.77	0.057	
**PERIPHERAL EMBOLISM**					
VKA	2				Ref.
FXa	2				0.37 (0.01–10.24)
DTI	2				0.55 (0.01–17.23)
Total	6	2.4%	1.36	0.50	

*VKA, vitamin K antagonist; FXa, Factor Xa inhibitor; DTI, direct thrombin inhibitor; CRNNMH, clinically relevant nonmajor hemorrhage. Cox regression analysis adjusted by sex, age, Charlson comorbidity index, and previous stroke*.

Overall, there were no significant differences for the main outcomes regarding the indication of anticoagulant therapy: stroke recurrences (13.3 vs. 21.4% vs. 7.9%; *p* = 0.411), intracranial hemorrhage (1.6% vs. 7.1% vs. 0%;*p* = 0.21), major bleeding (4.3% vs. 14.3% vs. 5.3%; *p* = 0.25), and all-cause mortality (21.3% vs. 21.4% vs. 31.6%; *p* = 0.38) for AF, prosthetic valves, and minor cardioembolic sources, respectively.

For secondary outcomes, we found a trend to a higher frequency of minor bleeding with VKA treatment (17.7% vs. 6.6% for FXa and 10% for DTI, *p* = 0.05). Among the 55 patients deceased during the follow-up, only five had a vascular-related cause of mortality; four of them were in the VKA group, and one was in the FXa group. Similarly, there were no differences in the long-term risk of secondary outcomes.

**Figure 5:**
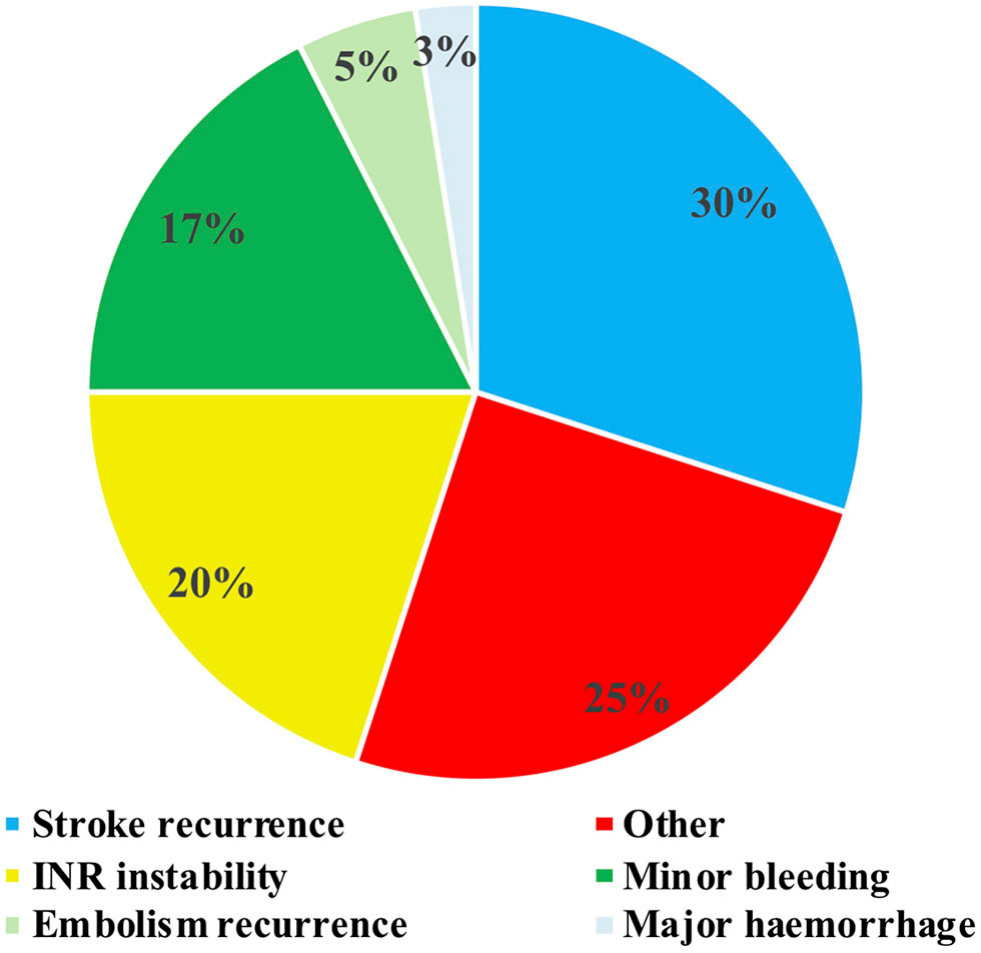
Reasons for anticoagulant switch during 3-years follow-up.

During the 3-years follow-up, none of the patients discontinued the anticoagulation therapy, although 40 switches to another anticoagulant were made; 62% were changed to FXa, 24.4% to DTI, and only 13.3% to VKA. The main reasons for changing were stroke recurrence (30%) and INR instability (20%), followed by minor bleeding (17%) (Figure 5). None of the patients changed the anticoagulant due to intracranial hemorrhage; the two survivor patients who had intracranial hemorrhage during follow-up were restored to the same DOAC (FXa) for long-term anticoagulation.

The average last INR reported at each follow-up visit in the VKA group was 2.6 (0.4 SD) at 3 months, 3.2 (1.1 SD) at 1 year, 1.5 (0.7 SD) at the second year, and 2.5 (0.7 SD) at the third year.

## Discussion

In this prospective registry that reflected routine clinical practice in secondary stroke prevention and included long-term follow-up, we found a higher cumulative incidence of stroke recurrence than of bleeding complications, without differences between the different OACs prescribed. Some prospective registries have analyzed the outcomes of patients on OAC in secondary stroke prevention, and they showed similar results, with no differences between VKA and DOACs regarding stroke recurrence (18, 33, 34). An individual patient data analysis of prospective studies recently found that VKA was associated with higher rates of mortality, intracranial hemorrhage, and combined vascular events compared with DOACs, but again, the rates for stroke recurrence were similar between groups (29).

Stroke patients represent a specific population in which the risk of intracranial hemorrhage should be especially taken into account (35, 36). In our study, we found a 17% cumulative incidence for recurrent stroke at a 3-years follow-up, which is comparable to other similar registries (17.5 and 18%) (4, 37). In our cohort, only four intracranial hemorrhages (1.6% cumulative incidence) were recorded at a 3-years follow-up, compared with 42 stroke recurrences. Similarly, in other registries, the rates of stroke recurrence were higher than those of intracranial hemorrhage (29, 38).

In our cohort, we did not find differences in the anticoagulation treatments for intracranial hemorrhage, in contrast to other registries that found a higher frequency of intracranial hemorrhage and fatal intracranial hemorrhage with VKA (29, 39). The smaller sample size and the good baseline functional status, with an average mRS of 1, of the patients included could explain this result. Nevertheless, our results also reinforce the idea of a higher risk of stroke recurrence than intracranial hemorrhage in secondary stroke prevention at long-term follow-up and, together with the MRI marker studies (27, 28), could improve the clinician decision about long-term anticoagulation in secondary prevention of stroke.

Patients with previous stroke before the index stroke presented a significantly higher rate of stroke recurrence during the follow-up. It is well-known that these patients are at higher risk of ischemic events even if on OACs, and similar findings have been previously reported (40). This should encourage us to look for better strategies to improve secondary stroke prevention, such as ensuring patients' adherence to treatment. The NOACISP-LONGTERM registry showed 78.4% of the patients to be fully adherent with a median follow-up of 12 months, and a recent systematic review showed that up to 30% of the patients with AF are non-adherent to any OAC (22, 41). Likewise, a Spanish study suggested that only 41.4% of the patients are treated following the current Spanish recommendations in clinical practice (42). In the Asian population, 18.8 and 13.1% of patients are either undertreated or overtreated, respectively, with a significant increase in adverse effects (43). Regarding the type of anticoagulant used, almost 50% of the patients treated with VKA are not well-anticoagulated, have diabetes, have labile INR, and have a high HAS-BLED score being associated with poor coagulation control (44). Although compliance is better with DOACs than with VKA, it has been reported that 13% of the AF patients receive DOAC doses that are inconsistent with labeling (9.4% underdosed and 3.4% overdosed) (45). This may reflect concerns about drug-related bleeding and may contribute to a higher stroke recurrence rate. Therefore, usage of appropriate dosage should be reinforced.

Regarding secondary outcomes, we found no statistically significant higher rates of minor bleeding with VKA treatment. In fact, up to 17% of the patients reported switching the anticoagulant drug due to minor bleeding. The concept of burden due to anticoagulation treatment measured by the Anti-Clot Treatment Scale (ACTS) (46) includes the perceived possibility of bleeding as a result of vigorous and daily activities. Recent studies found a strong association between patient dissatisfaction with OAC treatment and increased stroke risk (47, 48), probably due to lack of treatment adherence. In addition, another quality-of-life registry showed that patients on DOACs experienced higher satisfaction with oral anticoagulation than those on VKA (49). In this sense, the 2019 AHA/ACC/HRS guidelines for the management of patients with AF (50) recommend shared decision making between clinicians and patients for better adherence to treatment.

Another interesting finding in our study was the analysis of the switches to another anticoagulant. Before the index stroke, one third of the total cohort was on OAC, mostly VKA, and about 61% of them had INR values below the recommended range, consistent with other studies (21, 51). Therefore, in the majority of patients previously treated with anticoagulants, the index stroke was considered a therapeutic failure, and a change to a DOAC was indicated. During the long-term follow-up, the main reasons for a change of anticoagulant were stroke recurrence and labile INR, the majority of switches being from VKA to FXa. Current clinical guidelines recommend considering changing to another OAC if the patient had a stroke while already anticoagulated. However, the recommendation is weak and based on expert opinion (52). While an INR test is easily accessible for VKA, determination of DOAC activity is not available in daily clinical practice. It would be interesting to have a reliable test to anticipate the therapeutic failure and then adapt a therapeutic strategy. In this regard a recent study suggests that the activity of anticoagulation measured by specific DOAC plasma levels on admission is associated with stroke severity (53), and some studies support the reliability of DOAC determination (54, 55) especially in patients with acute ischemic stroke (56, 57). However, the ideal test is not yet widely available, and more studies are needed to guide future treatment decisions (58, 59).

The switch among anticoagulants could also reflect the changes in recent years in anticoagulant drug prescriptions, with the prescription of DOACs increasing in parallel with a decline in the use of VKA. DOACs are associated with higher adherence, easy use, fewer drug and food interactions, and lower rates of all-cause mortality and bleeding events (4). Its simple management without need of periodic ambulatory monitoring has gained importance in current times during the COVID-19 pandemic, suggesting another advantage of DOAC treatment for the future.

Our study has some limitations. First, the sample size was small, and there was a clear selection bias because during the inclusion period, recruiting centers participated in other competing observational studies and clinical trials on acute stroke and stroke prevention, a fact that could have limited the number of patients who were finally included in our registry; the study protocol did not include a registry of the possible candidates to participate in the study and the reason for the exclusion. Second, most of the patients had a good functional status at study entry, with a median mRS score of 1; therefore, our results may not be able to be extrapolated to disabled stroke patients. Third, we lack information on TTR for patients on VKA, information that would be very valuable for a deeper evaluation of the study outcomes and treatment adherence in that group. Instead, in order to obtain an approximated value of anticoagulation, we asked the participants to provide the information on the last INR value at each follow-up visit. The main strengths are the multicenter approach, the long-term follow-up (most published studies were carried out with 1- or 2-years follow-up), and the evaluation of multiple variables not usually included in the previously published registries (minor bleedings and anticoagulant switching during the follow-up and reasons for them).

## Conclusion

In this prospective hospital-based cohort, the rates of stroke recurrence in patients treated with anticoagulant drugs in a secondary prevention setting were higher than those of hemorrhagic complications, with no differences between the anticoagulant drugs. The majority of the changes between anticoagulants as a consequence of the index stroke and during the follow-up were from VKA to a DOAC, and the most common reason for change was stroke recurrence.

## Data Availability Statement

The raw data supporting the conclusions of this article will be made available by the authors, without undue reservation.

## Ethics Statement

The studies involving human participants were reviewed and approved by La Paz University Hospital ethics committee. The patients/participants provided their written informed consent to participate in this study.

## Author Contributions

RG-Z and RR: patients' follow-up, data management, data analysis, and writing the manuscript draft. GT-I, SS-V, MA, JM, RÁ, IN, LI-E, JF-F, JR-P, GR-A, and GZ-W: patients' recruitment, data collection, and critical review of the manuscript. BF and ED-T: study conception, study coordination, data interpretation, and critical review of the manuscript. All authors contributed to the article and approved the submitted version.

## Conflict of Interest

The authors declare that the research was conducted in the absence of any commercial or financial relationships that could be construed as a potential conflict of interest.
